# Enhancing El-Shohada distribution network: techno-economic optimization of PV and EV infrastructure using growth optimization algorithm

**DOI:** 10.1038/s41598-026-65285-y

**Published:** 2026-08-19

**Authors:** Adel A. Abou El-Ela, Eman Salah, Mohamed M. Hegab

**Affiliations:** Electric power systems, Electrical Engineering Department, Engineering faculty, Menoufia University, Shibīn al Kawm, Egypt

**Keywords:** Energy science and technology, Engineering, Mathematics and computing

## Abstract

This paper presents a comprehensive techno-economic framework for the optimal integration of distributed energy resources (DERs)—specifically Photovoltaic (PV) solar generation—and Electric Vehicle Charging Stations (EVCS) into modern electrical distribution grids. The methodology is applied to a realistic 25-bus, 11 kV radial distribution network modeled after the El-Shohada district in Menofia Governorate, Egypt. To balance inherently conflicting technical and financial objectives, a multi-objective penalty function is formulated. It simultaneously minimizes active power losses, mitigates voltage deviations, and maximizes investor profitability based on contemporary Egyptian energy tariff structures. The recently developed metaheuristic Growth Optimization (GO) algorithm is utilized to determine the optimal bus locations and capacities of these units over a 24-hour horizon, factoring in stochastic load profiles, solar irradiance, and EV charging behavior. Statistical validation based on 30 independent optimization runs demonstrates the high computational efficiency of the GO algorithm, yielding an optimal equilibrium that enhances grid reliability while providing a solid economic foundation.

## Introduction

 Modern electrical distribution networks are undergoing a massive paradigm shift due to the rapid deployment of distributed generation (DG) and the accelerating infrastructure of Electric Vehicles (EVs)^1–7^. While the integration of these technologies is crucial for carbon footprint reduction, their uncoordinated placement and sizing can severely degrade grid performance^8^. Random connection of large PV plants and EV charging stations often leads to severe technical complications, such as voltage sags during peak hours, excessive power losses in distribution lines, and accelerated thermal degradation of substation transformers^9,10^.

This study addresses these critical challenges by modelling a real-world case study: the 25-bus radial distribution network of the El-Shohada region operating at 11 kV^2,11^. The core objective is to frame a mathematical optimization problem solved via the state-of-the-art Growth Optimization (GO) algorithm^5^. The framework determines the absolute optimal.

nodes to integrate two distinct solar PV plants and two EV charging stations, alongside calculating their maximum design capacities to achieve a balanced trade-off between maximizing economic daily revenue and minimizing adverse grid impacts^3,12^.

The transition toward green energy ecosystems has forced a dual burden on regional distribution utilities: hosting volatile renewable energy injections while simultaneously feeding localized, high-power EV charging infrastructure^13,14^. In countries experiencing rapid infrastructure modernization, such as Egypt, this challenge is compounded by specific economic parameters, localized grid constraints, and distinct daily consumption behaviors^2,15^.

Uncoordinated EV charging loads act as heavy localized power drains, which typically peak during evening intervals when grid stress is already maximized^16^. Conversely, solar PV arrays supply power exclusively during daylight hours, creating a temporal mismatch^17^. If these entities are haphazardly connected to weak radial distribution lines—such as the 11 kV distribution networks found in peripheral regional zones like El-Shohada—they trigger severe voltage drops at remote buses and dramatically escalate line losses^11,18^.

Therefore, the core motivation of this research is to build an intelligent, multi-objective computational framework that bridges the gap between private investor ambitions and utility technical boundaries^19^. By modelling localized tariff rates, real capital expenditures (Capex), and stochastic environmental variations, this paper motivates the shift away from intuitive “rule-of-thumb” asset placement toward mathematically rigorous, metaheuristically driven grid optimization^6,20^.

### Knowledge gaps and methodological contributions

While various standard algorithms have been used for resource distribution, existing literature often oversimplifies localized network constraints, ignores practical multi-objective economic tariffs, or tests frameworks exclusively on idealized IEEE models. To address these gaps, the explicit technical contributions of this study are itemized as follows:

- Formulation of a highly specialized, multi-objective penalty framework tailored to localized grid codes and contemporary Egyptian energy tariff structures.
- Application of the advanced Growth Optimization Algorithm (GOA) to simultaneously resolve the spatial and capacity allocations of hybrid assets (2PV + 2EV) under highly non-linear constraints.
- Integration of continuous time-varying, 24-hour stochastic resource and demand profiling using superimposed Gaussian noise to assess system robustness.
- Empirical verification and statistical performance analysis over 30 independent optimization trials based on the real 11 kV El-Shohada regional distribution system.

### Literature survey

The problem of optimal allocation and sizing of Distributed Generation (DG) and Electric Vehicle Charging Stations (EVCS) has been extensively explored in recent power systems literature, utilizing various mathematical and metaheuristic techniques^1,21^.

Traditional optimization methods, such as mixed-integer linear programming (MILP) and analytical approaches, were initially favored for asset al.location due to their deterministic nature^22^. However, as highlighted by continuous power grid evolutions, these classical techniques struggle significantly when dealing with the highly non-linear, non-convex constraints inherent in multi-bus radial distribution load flows^23^. Consequently, the academic consensus has shifted heavily toward metaheuristic optimization algorithms^24^.

Several swarm intelligence and evolutionary algorithms have been deployed to mitigate grid vulnerabilities. For instance, the Particle Swarm Optimization (PSO) algorithm has been widely used to determine optimal DG placement to reduce active power losses^25^. While PSO exhibits fast convergence, it frequently suffers from premature convergence, trapping the solution in local minima when exploring multi-dimensional search spaces containing both discrete bus locations and continuous capacity variables^26^. Similarly, the Genetic Algorithm (GA) has been utilized for sizing EV charging hubs; however, GA requires heavy computational overhead and complex mutation/crossover tuning parameters to maintain population diversity over dynamic 24-hour cycles^27^.

Recent literature has focused heavily on co-allocation strategies, recognizing that the reactive and active power profiles of EVCS and solar DGs can complement each other if placed strategically^14,28^. Algorithms such as the Grey Wolf Optimizer (GWO), Moth-Flame Optimization (MFO), and the Harris Hawks Optimization (HHO) have been adapted for hybrid renewable-EV grid planning^29^. While these algorithms show improved exploratory capabilities, they often require complex parameter tuning and can exhibit slow convergence characteristics during high-dimensional grid operations^30–49^.

To overcome these computational bottlenecks, this paper introduces the application of the Growth Optimization (GO) algorithm to the co-allocation problem^5^. GO mimics natural growth, social learning, and environmental reflection mechanisms, striking an elite balance between exploration (global search) and exploitation (local refinement)^5,6^. Furthermore, while most existing literature evaluates these networks using standard, idealized IEEE test systems (e.g., IEEE 33-bus or 69-bus networks)^13,25^, this study contributes to the literature by applying the optimization framework to a realistic, locally modelled 25-bus regional distribution network using real-world Egyptian market pricing, operational constraints, and stochastic weather noise^2,4^ (Table 1).

**Table 1:** Comparative summary matrix of prior literature versus the proposed framework.

Reference	Core methodology	Test system	Objectives considered	Operational limitations addressed
Carrión	Comprehensive Review	Standard Systems	Asset Sizing & Siting	General review baseline
Atwa	MILP	Radial Networks	Mix of Renewable Units	Deterministic, non-stochastic
El-Zonkoly	PSO Variants	IEEE Layouts	Loss Reduction & Profile Sizing	Prone to local optima traps
Ahmed	Voltage Indices	Stochastic Loads	Voltage Drop Tracking	No economic metaheuristic allocation
Proposed Work	GOA Framework	Real 25-Bus Grid	Loss, Voltage & Net Profit	Stochastic multi-objective co-allocation

## Methods

### Mathematical modeling of the system

#### Per-unit system conversion

To standardize variables across different orders of magnitude and simplify network equations, the entire distribution network dataset is transformed into the per-unit (p.u.) system^23^. The base values are established as follows:

- Base Power (S_base_): 1 MVA.
- Base Voltage (V_base_): 11 kV.
- Base Impedance (Z_base_):

1 $$Zbase = \frac{{Vbase^{2} }}{{Sbase}} = 121\,ohm$$

Line resistances (R) and reactances (X) are divided by Z_base_, while the base active (P_L_) and reactive (Q_L_) loads at each bus are scaled by S_base_^11^.

#### Nodal admittance matrix (Y_base_) construction

The physical configuration and electrical relationships of the 25-bus network are mathematically structured using the nodal admittance matrix (Y_base_)^23^. The elements of this matrix are formulated through the following standard branch equations:

- Self-admittance (Diagonal Elements):

2 $$Y_{{ii}} = \mathop \sum \limits_{{j \in \Omega _{i} }} y_{{ij}} ~~~~~~$$

- Mutual admittance (Off-Diagonal Elements):

Represents the complex admittance of the feeder section connecting bus i to bus j^11^.

3 $$Y_{{ij}} = y_{{ij}}$$

where

4 $$y_{{ij}} = \frac{1}{{R_{{ij}} + jX_{{ij}} }}~~~~~$$

#### 24-Hour stochastic resource and demand profiling

Grid behavior fluctuates dynamically throughout the day^16^. To mirror real-world uncertainties, three time-varying, 24-hour profiles are implemented in the model:

1. Load profile (load_profile): Represents typical human and commercial electricity consumption trends in El-Shohada, peaking at Hour 18 (100%)^2^.
2. Solar generation profile (solar_profile): Models solar irradiance from sunrise to sunset, peaking at noon^17^. To simulate unpredictable cloud coverage and weather anomalies, a 5% Gaussian noise component is superimposed^12,31,32^:

5 $$Solar{\text{ }}\left( t \right){\text{ }} = {\text{ }}Solar_{{base}} \left( t \right){\text{ }}x{\text{ }}(1{\text{ }} + {\text{ }}0.05\mu N{\text{ }}\left( {0,{\text{ }}1} \right))$$

3. EV charging profile (ev_profile): Replicates the power demand pattern of EV charging facilities, which experiences its highest utilization in the evening as vehicles return from daily commutes^8^. A 5% random Gaussian noise is integrated to represent behavioral uncertainty^16^:

6 $$EV{\text{ }}\left( t \right){\text{ }} = {\text{ }}EV_{{base}} \left( t \right){\text{ }}x{\text{ }}(1{\text{ }} + {\text{ }}0.1\mu N{\text{ }}\left( {0,{\text{ }}1} \right))$$

N (0, 1) denotes a standard normal distribution with a mean of zero and a variance of one.

#### Gauss-Seidel load flow engine

To evaluate the voltage magnitude and phase angle at every single node across the 24-hour horizon, a dynamic Gauss-Seidel load flow computational engine was 0developed^3,11^. While several distribution-specific load flow techniques, such as the Backward/Forward Sweep (BFS) method, are conventionally applied to radial topologies, the dynamic Gauss-Seidel (GS) load flow engine is intentionally adopted in this study. The primary rationale is the highly non-linear, stochastic, and bidirectional nature of contemporary distributed energy resources (DERs)—specifically the multi-period continuous active and reactive power profiles injected or absorbed by the EVCS units. The GS algorithm offers seamless matrix formulation compatibility when embedding these fast-changing, time-variant algebraic node injections into the net active and reactive power equations P^net^_i_ and Q^net^_i_ at each iteration without necessitating structural modifications to the branch path dependency matrices. To guarantee absolute mathematical precision and overcome any potential divergence risks associated with high R/X ratios in weak radial lines, a strict convergence tolerance criterion (${\mathrm{\boldsymbol{\upvarepsilon}}}$= 10^− 6^ p.u.) was strictly enforced across all 24-hour simulation periods. The nodal voltages are iteratively updated using the following non-linear relationship:

7 $$V^{{(new)}} \frac{1}{{y_{i} }}~\left[ {~\frac{{P_{i}^{{net}} - jQ_{i}^{{net}} }}{{(V_{i}^{{\left( {old} \right)}} )*}} - ~\Sigma _{{j = i}} Y_{i} j~V_{j} } \right]$$

The net injected active and reactive powers (P^net^_i__, Q^net^_i_) at any given bus i are continuously adjusted based on the optimal placement of the allocated units^28^:

With Solar PV connected:

8 $$P^{{net}} _{i} = {\text{ }}P_{{load}} \left( t \right){\text{ }}{-}{\text{ }}P_{{PV}} \left( t \right)~$$

With EVCS connected:

9 $$P^{{net}} _{i} = {\text{ }}P_{{load}} \left( t \right){\text{ }} + {\text{ }}P_{{EV}} \left( t \right)$$

10 $$Q_{i}^{n} et = Qload(t) + PEV(t)*\tan (\cos - 1(0.95))$$

To evaluate the voltage magnitude and phase angle at every single node across the 24-hour horizon, a dynamic Gauss-Seidel load flow computational engine was developed^3,11^. The net injected active power (P^net^_i_) at any given bus i is continuously adjusted based on the optimal placement of the allocated units^28^:

11 $$P^{{net}} _{i} = P_{{load}} \left( t \right) + P_{{EV}} \left( t \right) - P_{{PV}} \left( t \right)$$

#### Optimization problem formulation

##### Decision variables

The multi-dimensional search space consists of 8 decision variables (Dim = 8) vectorized as follows^24^:

12 $$X = [Bus_{{PV1}} ,{\text{ }}Bus_{{PV2,}} Bus_{{EV1,}} Bus_{{EV2,}} Size_{{PV1,}} Size_{{PV2,}} Size_{{EV1,}} Size_{{EV2}} ]~~~$$

##### Bounds and constraints

- Location boundaries:
  13 $$2{\text{ }} \le {\text{ }}Bus{\text{ }} \le 25$$
  (Bus 1 is designated as the Slack/Substation bus)^11^.
- Solar PV sizing limits:
  14 $$150{\text{ }} \le Size_{{PV}} \le 2000{\text{ }}kW$$
  (optimized in discrete steps of 50 kW)^4^.
- EVCS sizing limits:
  15 $$100{\text{ }} \le Size_{{EV}} \le 1500{\text{ }}kW$$
  (optimized in discrete steps of 50 kW)^19^.

##### Multi-objective function derivation

The objective is to minimize a holistic penalty index (F(**X**)) that weights technical violations against financial deficits^3,30^.

16 $$\begin{aligned} Minimize{\text{ }}\left( {F\left( X \right)} \right){\text{ }} & = {\text{ }}\left( {W_{{Loss}} x{\text{ }}P_{{Loss - Total}} } \right){\text{ }} + {\text{ }}\left( {W_{{Voltage}} x{\text{ }}\Delta V} \right){\text{ }} \\ & + {\text{ }}\left( {W_{{\cos t}} x{\text{ }}Net\_Daily\_Loss} \right)~~~~~~ \\ \end{aligned}$$

Where:

1. Total active power losses:

17 $$PLoss - Total = \sum _{{(i = 1)}}^{{24}} P_{{loss}} (t)$$

(Weight coefficient W_Loss_ = 2.0)^3^.

2. Voltage deviation index:

18 $$\Delta V = \mathop \sum \limits_{{i = 1}}^{{24}} \mathop \sum \limits_{{i = 1}}^{{Nbus}} (1.0 - |V_{i} \left( t \right)|)$$

(Weight coefficient W_Voltage_= 1000.0)^10^.

3. Net daily economic loss:

(Weight coefficient W_Cost_= 0.1)^12^.

19 $$Net\_Daily\_Loss{\text{ }} = {\text{ }}Daily\_\mathrm{Cos} t{\text{ }}{-}{\text{ }}Total\_Daily\_\mathrm{Re} venue$$

##### Financial modeling parameters

- Capital expenditure (CapEx): The investment cost for Solar PV is modeled at 27,000 EGP/kW, while EV charging infrastructure is estimated at 18,000 EGP/kW^4^.

Amortized Daily Cost (Daily_Cost): Based on a project lifecycle of 20 years, combined with an annual Operation and Maintenance (O&M) rate equal to 2% of the total CapEx^12^.

20 $$Daily\_\mathrm{Cos} t = \frac{{Total\_CAPEX}}{{20*365}} + \frac{{Total\_CAPEX*0.02}}{{365}}$$

- Revenue streams: The tariff for selling solar-generated electricity back to the grid is set at 1.45 EGP/kWh, while the commercial charging price billed to EV consumers is structured at 5.00 EGP/kWh^4^.

#### Growth optimization algorithm

**Fig. 1:**
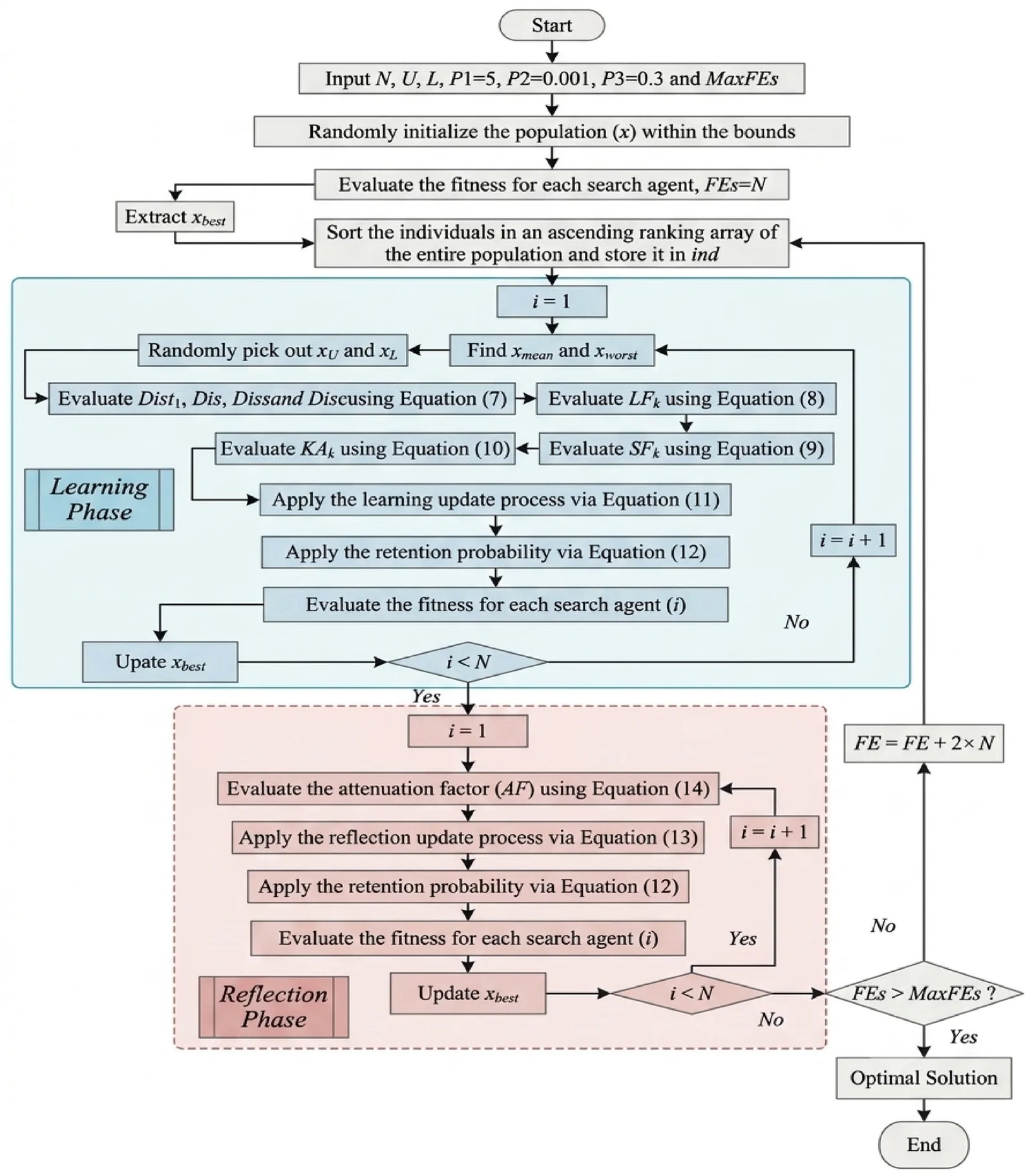
Flowchart of the GO algorithm.

To resolve the highly non-linear, multi-objective allocation problem, the framework employs the Growth Optimization (GO) algorithm. Unlike traditional metaheuristics such as GWO, HHO, or MPA^32–49^, which often experience stagnation due to rigid mathematical searching steps, GOA models social growth, learning, and self-reflection mechanics. This ensures an elite balance between global exploration (Learning Phase) and precise localized exploitation (Reflection Phase), providing an efficient computational path to global minima without trapping variables in discrete local optima. The mathematical population consists of a designated number of individual parameter sets (candidate solutions). The optimization procedure operates recursively through two major iterative phases as shown in the systemic flow chart (Fig. 1):

### The learning phase

During the learning phase, candidate solution vectors interactively update their positions by closing spatial gaps relative to the top-performing individuals (the elite parameter boundaries) and the global leader (Xbest).

1. Gap recognition: Each search agent maps its relative distance to the absolute best solution and lower-ranked baseline solutions to deduce profitable search directions.
2. Knowledge assimilation: The location of the agent is updated via a vector step process where learning factors (LF) and knowledge acquisition (KA) indices govern the movement step size towards or away from neighboring solutions.
3. Retention probability evaluation: Changes are subjected to a historical retention probability criterion to check if the newly acquired state yield’s superior objective evaluation compared to the previous state.

### The reflection phase

To prevent premature convergence and enhance exploratory behavior, the population undergoes a self-reflection mechanism mimicking individual retrospection.

1. Inefficacy mitigation: If an individual search agent determines that its parameter convergence has stalled or fallen behind the global trend, it modifies its search vectors.
2. Attenuation scaling: An attenuation factor (AF) is dynamically computed to scale down large step sizes, allowing fine-tuned, localized exploitation of the solution space.
3. Exploratory adjustment: Agents with insufficient knowledge are re-randomized or aggressively adjusted against the global best parameters to maintain population diversity, ensuring robust convergence toward global optimal solutions for active branch line loss reduction and optimal load shifting.

## Sampling plan

### Applications

#### Test systems under study

**Fig. 2:**
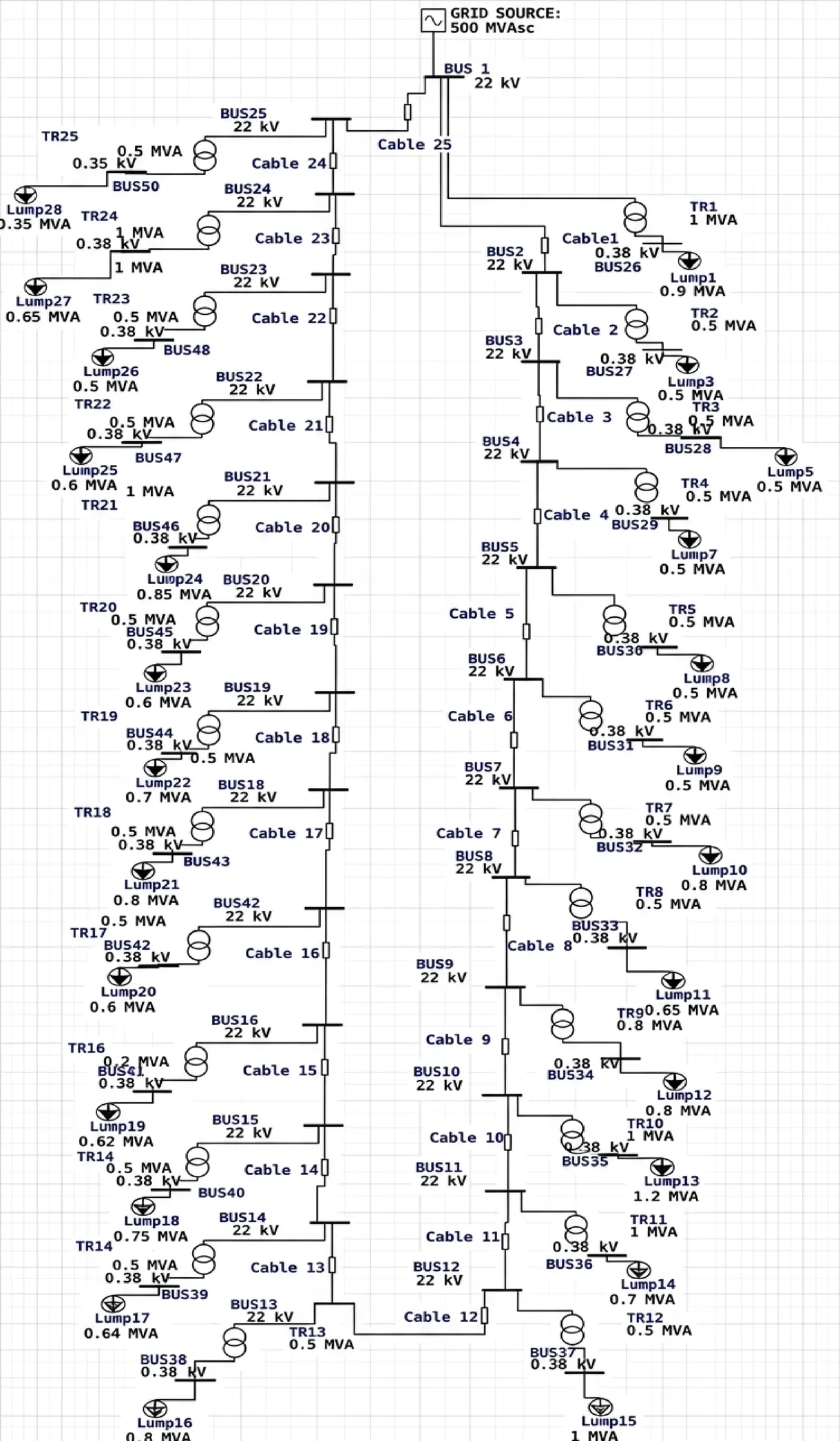
A real distribution system 25 buses in the south delta network.

The GO algorithm is used for a distribution grid a system of 25-bus system which is real network in the south delta network as an actual compartment of the Egyptian distribution grid as shown in Fig. 2. The total active and reactive loads of this real grid are 15.09 MW and 9.345 MVAR, respectively. The initial power losses are 1.831 MW.

**Fig. 3:**
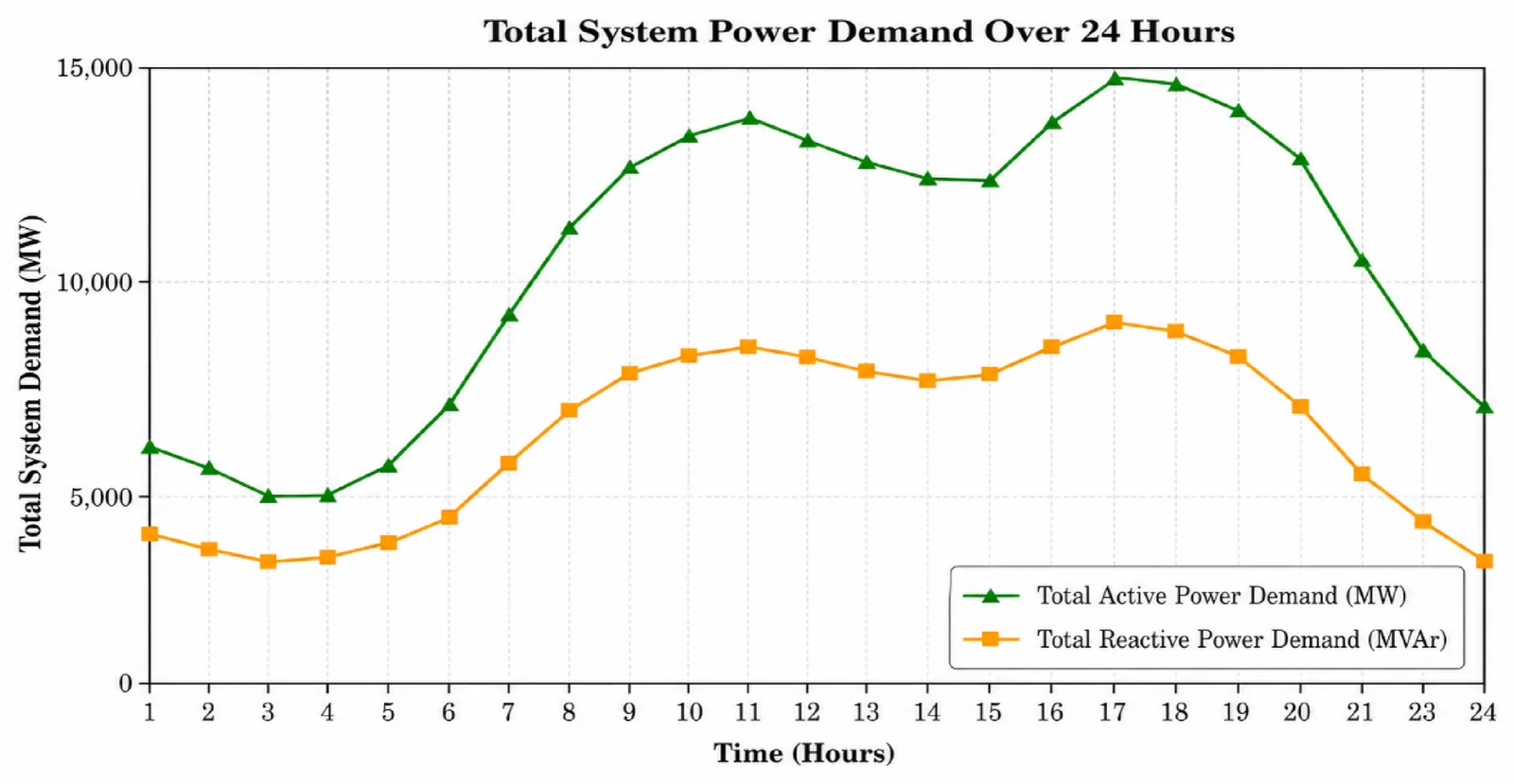
System loads active and reactive power profile with maximum load demand among 24 h.

Figure 3 shows the active and reactive power profile of 25 busloads system. The power is varying for each load among 24 h with respect to maximum load profile.

**Fig. 4:**
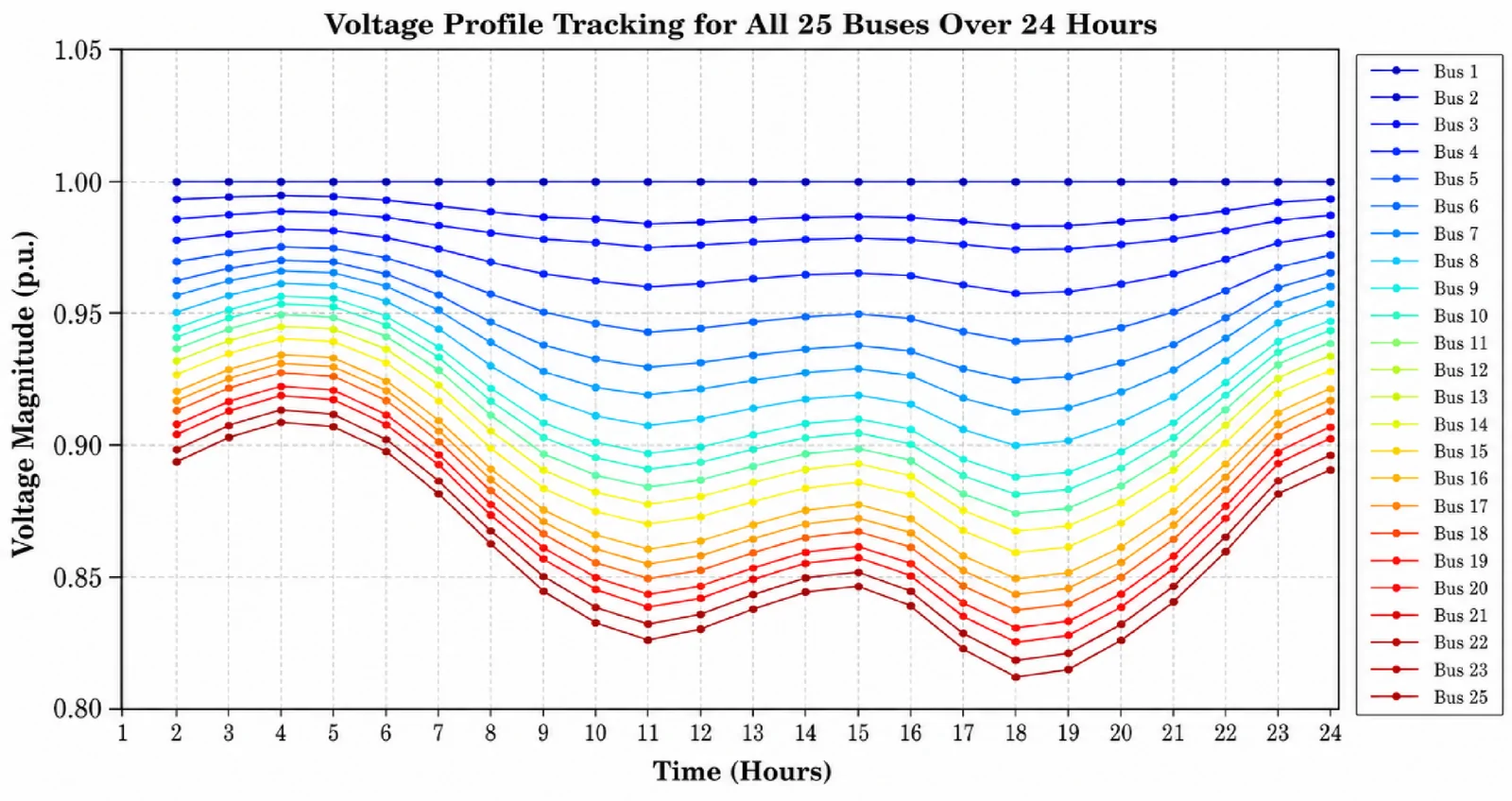
System voltage profile of 25 busses.

Figure 4 shows the voltage profile of 25 busloads at the full power of the system. The minimum voltage of the system is 0.80 P.U. at bus 14 at 18 PM.

**Fig. 5:**
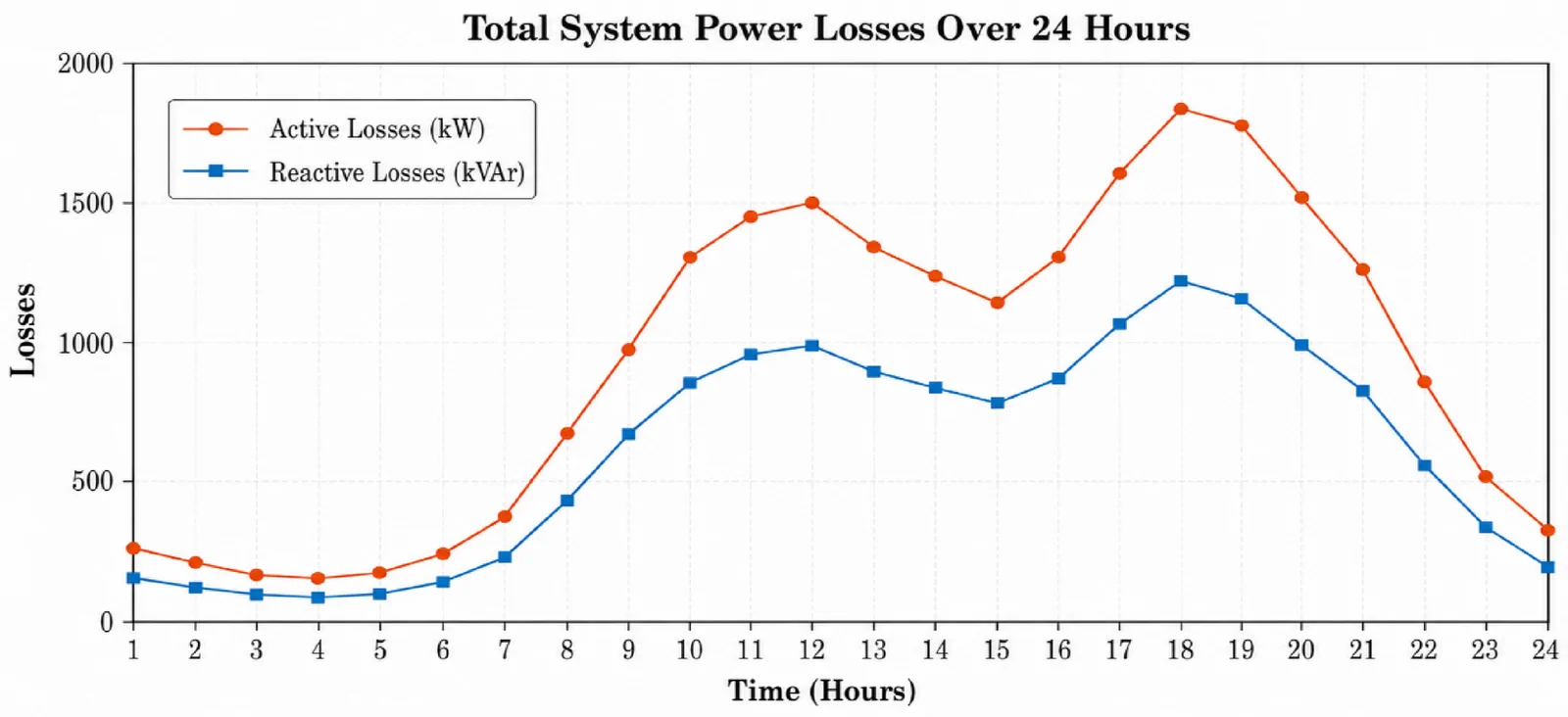
System active and reactive losses profile with maximum load demand among 24 h.

Figure 5 shows the system active and reactive losses profile of 25 busloads at the full power of the system. The maximum power active and reactive loss of the system are 1.831 MW and 1.194 MVAR at 18 PM.

## Simulation results and discussion

After studying the technical load flow parameters of the real case study system and the results are recorded after using the GOA technique to reach the best solutions at three scenarios, which are:

System enhancement using two PV stations only.

System enhancement using two EV stations only.

System enhancement using two PV and two EV stations.

### Optimal asset allocation and financial feasibility

#### For system enhancement using two PV stations only

**Fig. 6:**
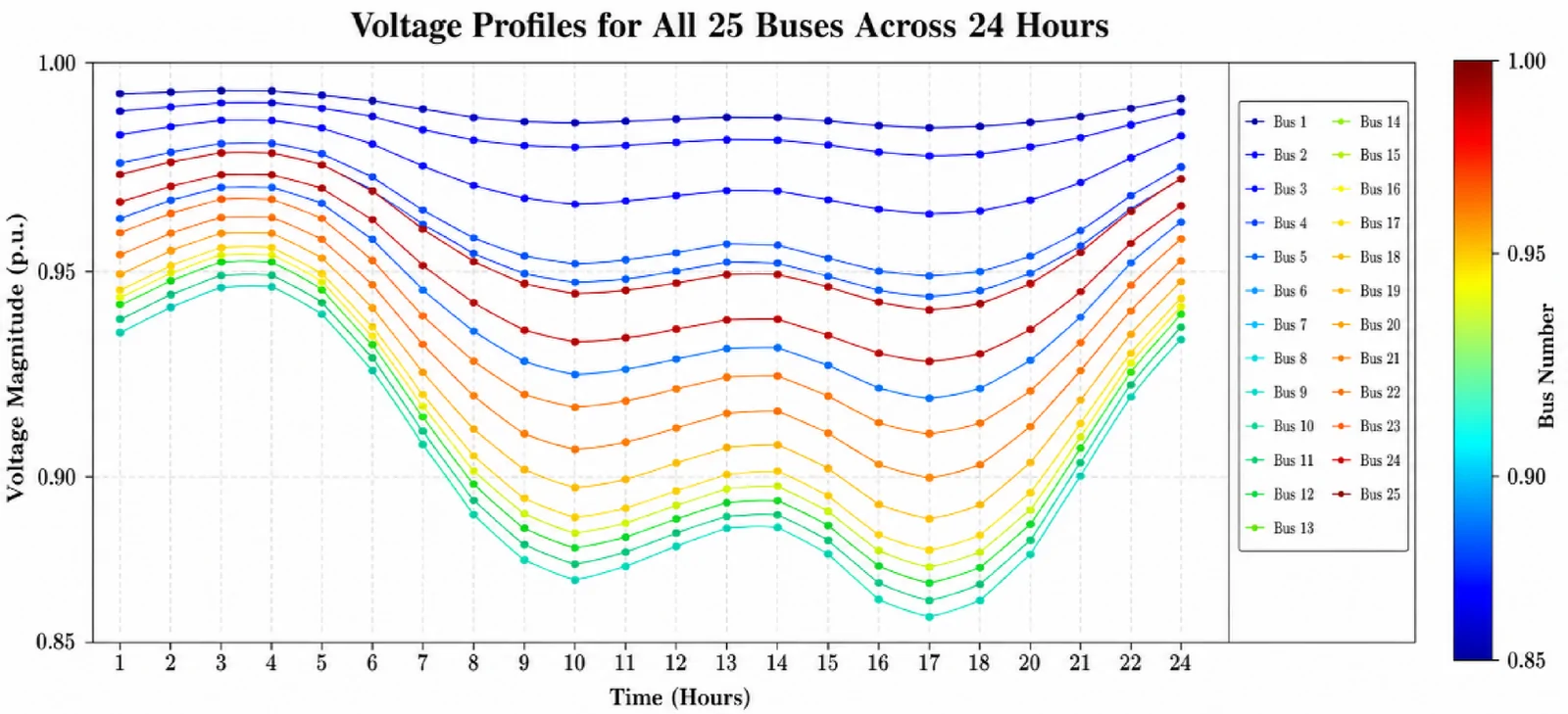
Voltage profile of 25 busses among 24 h after enhancement using two PV stations only.

Figure 6 illustrates the daily voltage profiles for all 25 buses within the distribution network over a 24-hour simulation period, specifically considering the case of PV power plant integration alone. The voltage magnitudes are expressed in per-unit (p.u.) values across the time horizon.

A distinct voltage dip is observed across the sensitive buses (represented by the warmer green and yellow color spectrums) during the late morning, reaching a local minimum around Hour 11. However, due to peak solar irradiance, a noticeable voltage recovery/peak occurs between Hour 12 and Hour 15, where the voltage magnitudes increase significantly (e.g., rising back to nearly 0.88 p.u. at Hour 15 for the lowest-performing buses). This highlights the localized reactive and active power support provided by the PV units during peak generation hours. Following the decline of solar generation, a severe voltage sag is observed during the evening peak load hours, reaching its absolute minimum at Hour 18. At this critical period, several remote buses experience severe under voltage conditions, dropping to approximately 0.838 p.u., which falls well below the standard permissible operational limit of pm5(i.e., p.u.). In conclusion, while the integration of solar PV units provides effective voltage support during daylight hours (Hours 12–15), it is insufficient to mitigate the severe under voltage issues during the evening peak (Hour 18). This demonstrates the absolute necessity for incorporating complementary grid assets, such as EV to ensure comprehensive voltage regulation across the entire 24-hour cycle.

**Fig. 7:**
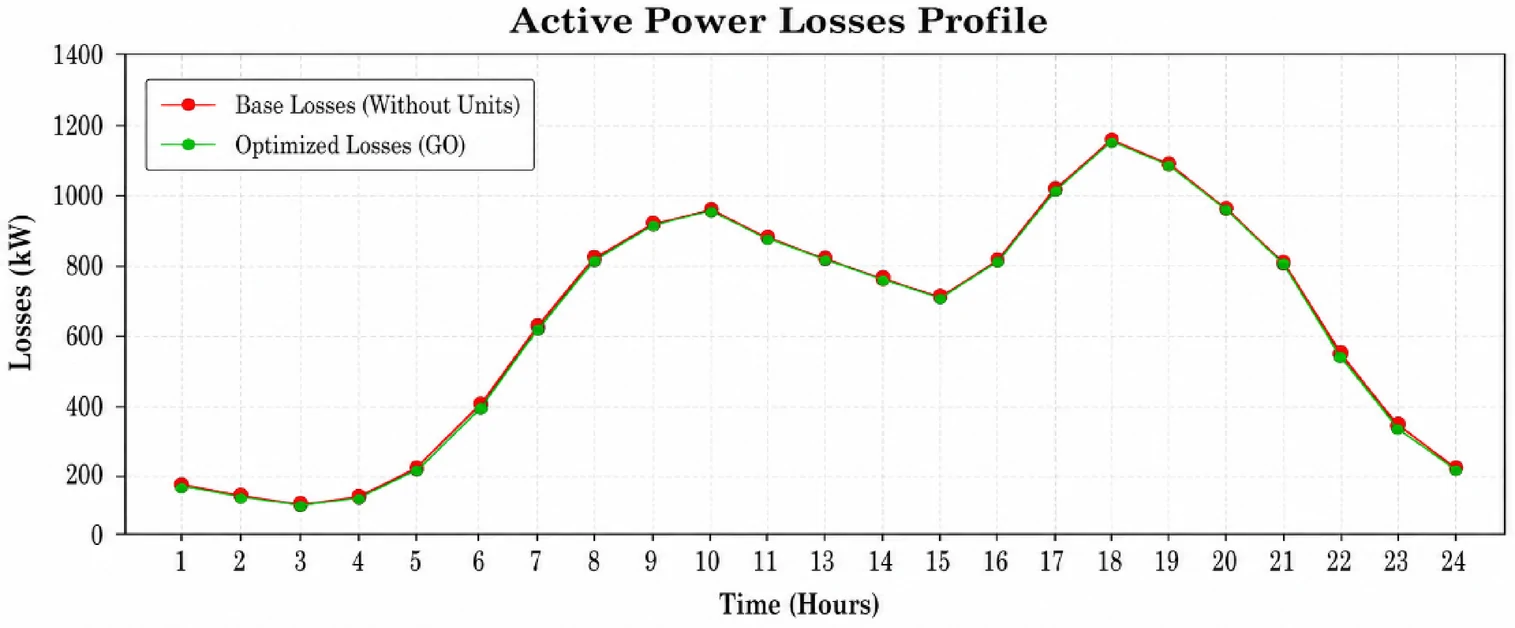
Power losses profile of 25 busses among 24 h after enhancement using two PV stations only.

Figure 7 portrays the 24-hour profile of the network’s active power losses (kW) under the scenario involving solar PV plants only. The graph compares the “Base Losses (Without Units)” represented by the red circular markers against the “Optimized Losses (GO)” represented by the green curve.

During the off-peak daylight hours, the network experiences a noticeable local peak in losses at Hour 11, reaching approximately 960 kW. However, a distinctive dip in active power losses is observed between Hour 12 and Hour 15, dropping to a local minimum of roughly 700 kW at Hour 15. This localized reduction directly correlates with peak solar irradiance, demonstrating that the active power injected locally by the PV units successfully reduces the upstream transmission line loading and associated I^2^ R losses during peak generation hours.

Table 2 presents the optimization results for the allocation, sizing, and economic metrics of the PV energy stations within the 25-bus distribution network. The optimization framework evaluates the spatial placement alongside the corresponding capacity requirements and capital investments.

**Table 2:** PV allocations and CAPEX.

Unit_Type	Optimal_Bus	Capacity_kW	Capital_Cost_EGP
PV Solar 1	8	2000	5.40E + 07
PV Solar 2	20	2000	5.40E + 07

**Fig. 8:**
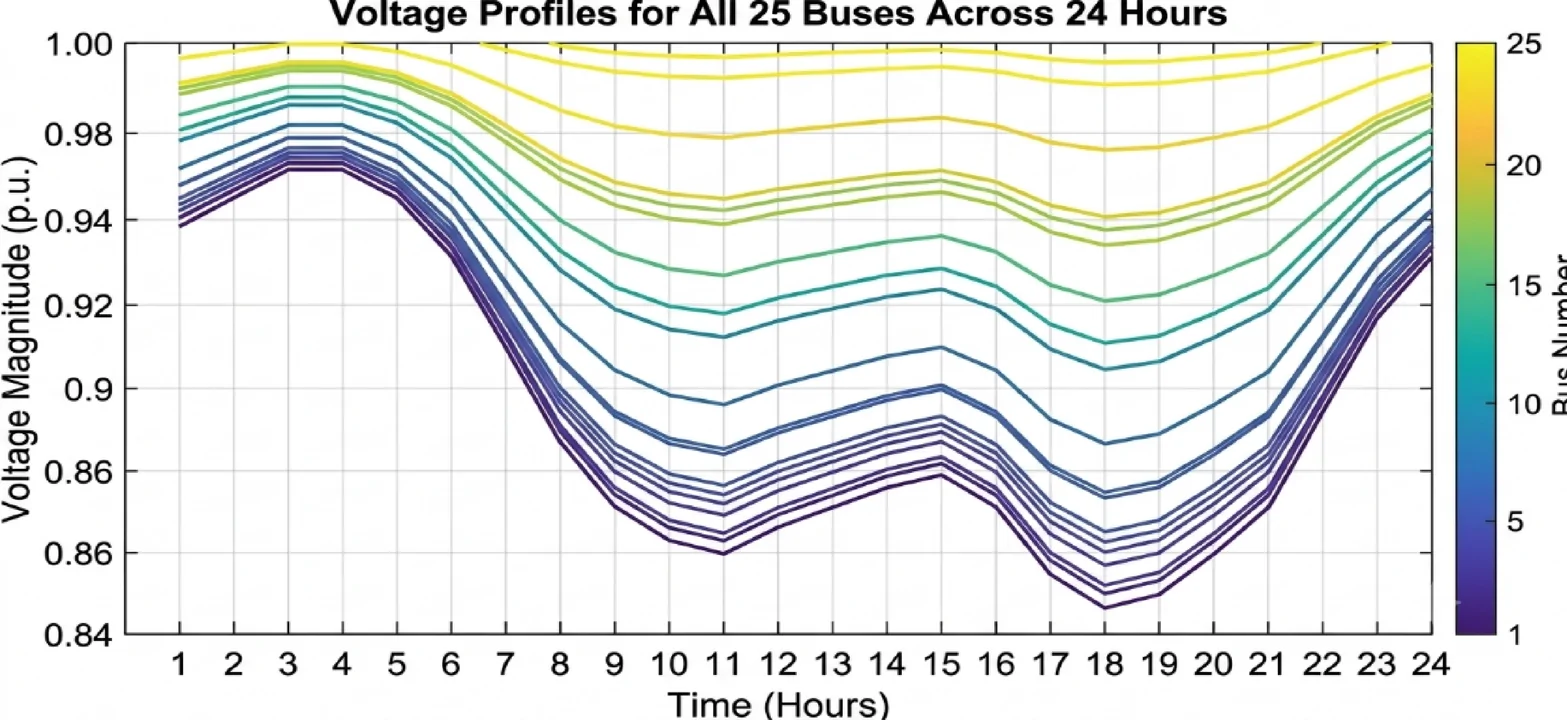
Voltage profile of 25 busses among 24 Hours after enhancement using two EV stations only. The voltage profiles across the vulnerable downstream buses (highlighted by the yellow and green color spectrums) display a clear "double-dip" phenomenon. The first significant drop occurs at Hour 11, where voltages sink to approximately 0.86 p.u. A minor, temporary recovery is observed between Hours 12 and 15, peaking around 0.88 p.u., which corresponds to a mid-day reduction in typical residential/commercial base load.

Figure 8 demonstrates the 24-hour daily voltage profiles for all 25 buses within the distribution network under the scenario considering the integration of Electric Vehicle (EV) charging stations only. The voltage magnitudes across the network are evaluated in per-unit (p.u.) values over the 24-hour time horizon.

#### For system enhancement using two EV stations only

The most severe system degradation occurs during the evening hours, culminating in an absolute minimum voltage drop at Hour 18. At this critical point, the uncoordinated charging load of EVs heavily coincides with the standard daily peak demand, driving the voltage of the most remote buses down to a critical nadir of roughly 0.832 p.u. This severe violation falls drastically below the standard grid operational threshold of 0.95 p.u., posing a major risk of localized voltage collapse.

**Fig. 9:**
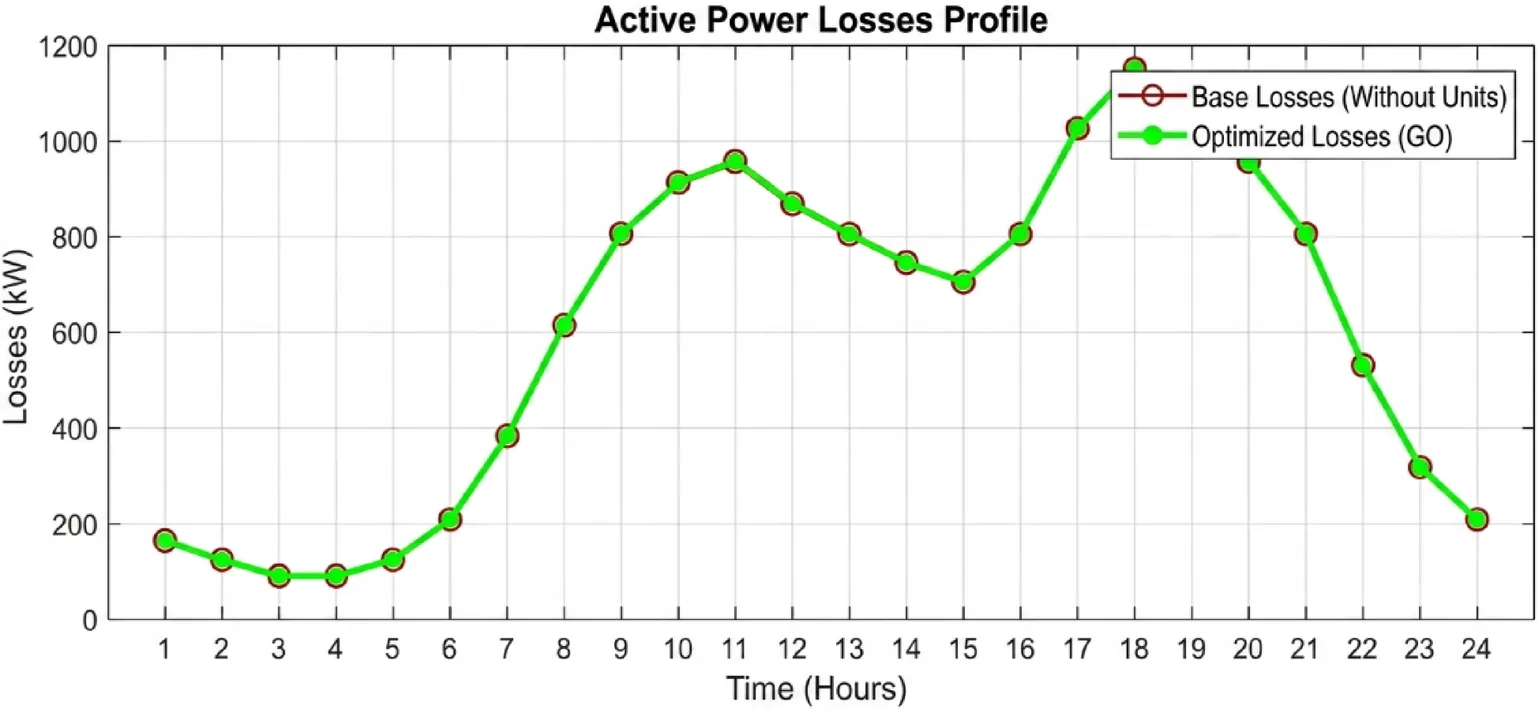
Power losses profile of 25 busses among 24 h after enhancement using two EV stations only.

Figure 9 demonstrates the 24-hour daily active power losses (kW) profile under the specific scenario of Electric Vehicle (EV) charging station integration alone. The figure tracks and compares the “Base Losses (Without Units)” depicted by the red circular indicators against the “Optimized Losses (GO)” plotted as a continuous green line.

System losses increase steadily during the morning hours, peaking locally at Hour 11 with a loss value of approximately 960 kW. This is followed by a temporary reduction in losses between Hour 12 and Hour 15, dropping to a local trough of roughly 700 kW at Hour 15. This midday decline is driven by the structural dip in typical residential base loads before the evening peak.

In summary, treating EV charging stations as passive, rigid loads lead to substantial active power losses, especially during peak grid hours (Hour 18). Because the optimization algorithm cannot independently alter this load profile in the absence of control strategies, the optimized trajectory remains identical to the base losses. These results emphasize that expanding EV hosting capacity without smart charging controls, Vehicle-to-Grid (V2G) scheduling, or local energy storage integration will drastically increase system losses and compromise grid efficiency.

Table 3 presents the optimization results for the allocation, sizing, and economic metrics of the Electric Vehicle (EV) charging stations within the 25-bus distribution network. The optimization framework evaluates the spatial placement alongside the corresponding capacity requirements and capital investments.

**Table 3:** EV allocations and CAPEX.

Unit_Type	Optimal_Bus	Capacity_kW	Capital_Cost_EGP
EV Charging 1	2	1500	2.70E+07
EV Charging 2	25	1500	2.70E+07

#### System enhancement using two PV and two EV stations

**Fig. 10:**
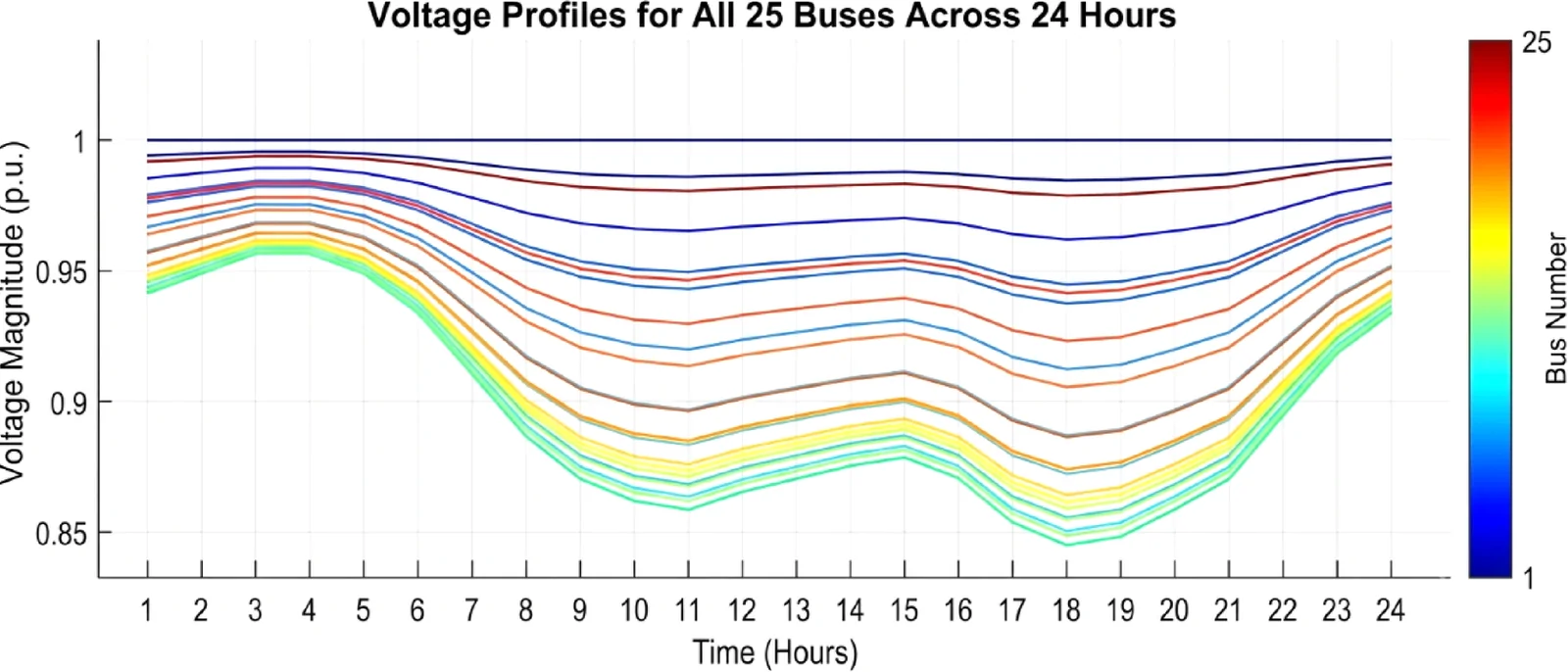
voltage profile of 25 busses among 24 h after enhancement using two PV & two EV stations. Between Hour 12 and Hour 15, a distinct upward inflection in the voltage profiles is visible across all peripheral nodes (yellow and green curves), peaking at Hour 15 where the lowest bus voltages recover to approximately 0.88p.u. This localized recovery highlights the technical benefit of matching the peak active power injection from the PV units with the midday distribution demand, effectively pushing the system away from its morning drop at Hour 11 (0.86 p.u.).Despite the midday active power support from the solar units, the grid experiences severe under voltage conditions during the evening peak. At Hour 18, because solar irradiance drops to zero while the uncoordinated EV charging load coincides with the peak residential demand, the voltage magnitudes of the most vulnerable downstream buses plunge to an absolute nadir of approximately 0.845 p.u. This represents a critical violation well below the standard technical tolerance threshold of 0.95 p.u.

Figure 10 illustrates the 24-hour daily voltage profiles across all 25 network buses under the co-allocation scenario of solar power plants and electric vehicle charging stations (2PV + 2 EV), representing the shared hybrid connection configuration. The voltage magnitudes are measured in per-unit (p.u.) over the complete diurnal cycle.

In summary, the joint integration of 2PV + 2 EV under an uncoordinated shared hybrid framework demonstrates that real-time solar generation is fundamentally decoupled from the evening peak charging demand. While the PV installations successfully cushion the network from deeper voltage drops during peak daylight hours, they provide no relief during the critical evening surge at Hour 18. This underscores that simply co-locating solar and EV infrastructures is insufficient to ensure grid compliance; implementing intelligent energy storage system (ESS) scheduling or dynamic demand-side management is mandatory to transfer the midday solar surplus to the critical evening valley.

**Fig. 11:**
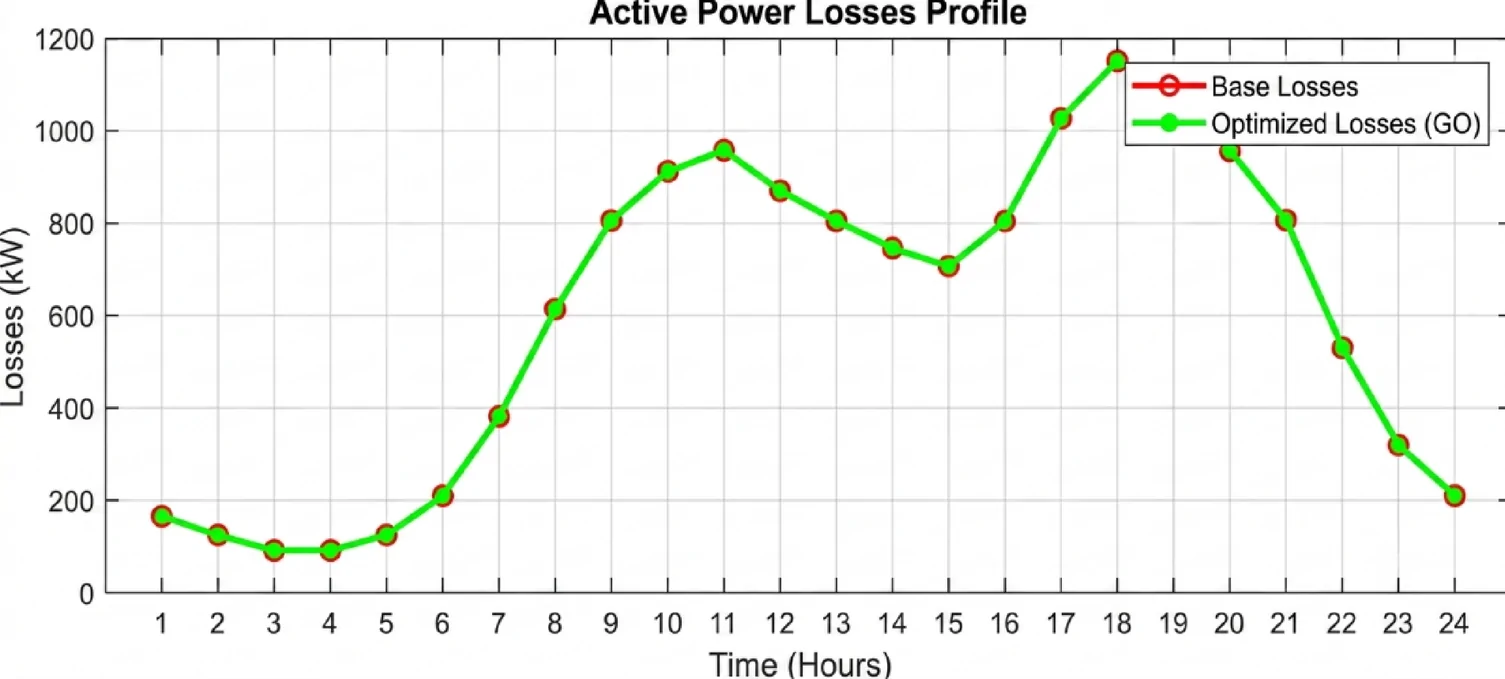
Power losses profile of 25 busses among 24 h after enhancement using two PV & two EV stations.

Figure 11 illustrates the 24-hour daily active power losses (kW) profile under the co-allocation scenario of solar power plants and electric vehicle charging stations (2PV} + 2 EV), reflecting the shared hybrid connection scheme. The figure tracks and contrasts the baseline performance (“Base Losses (Without Units)”, indicated by red circles) with the optimized performance managed by the algorithm (“Optimized Losses (GO)”, plotted as a continuous green line).

During the diurnal period, active power losses rise steadily in the morning to a local peak of approximately 960 kW at Hour 11. However, between Hour 12 and Hour 15, a clear drop in line losses occurs, sinking to a local valley of roughly 700 kWat Hour 15. This localized reduction demonstrates the beneficial impact of peak solar active power generation, which satisfies part of the heavy load demand locally and reduces the upstream feeder loading and associated I^2^ R ohmic losses.

Following sunset, the distribution system experiences severe thermal and loading stress. Due to the complete cessation of PV power generation coinciding with the highest daily consumer base load and uncoordinated evening EV home-charging, active power losses surge exponentially to an absolute maximum of approximately 1150 kW at Hour 18.

Table 4 presents the optimization outcomes for the simultaneous allocation, sizing, and capital investment requirements of the hybrid distributed network, consisting of Photovoltaic (PV) solar plants and Electric Vehicle (EV) charging stations. The multi-objective framework determines the optimal spatial node placement alongside capacity configurations to maximize grid efficiency under economic constraints.

**Table 4:** PV& EV allocations and CAPEX.

Unit_type	Optimal_Bus	Capacity_kW	Capital_Cost_EGP
PV Solar 1	**2**	**1750**	**4.725E+07**
PV Solar 2	**4**	**650**	**1.755E+07**
EV Charging 1	**2**	**1500**	**2.70E+07**
EV Charging 2	**12**	**1500**	**2.70E+07**

Analysis of Table 5 clearly indicates that the proposed Growth Optimization Algorithm (GOA) outperforms both GA and PSO across all technical benchmarks. GOA achieved a reduction in active power losses to 1112.4 kW, while maintaining the tightest voltage profile deviation. Furthermore, its computational efficiency is demonstrated by a significantly lower convergence time of 18.6 seconds, proving its suitability for real-time smart grid management.”

**Table 5:** Statistical optimization profile for GOA.

Optimization algorithm	Total active power losses (kW)	Maximum voltage deviation (p.u.)	Execution convergence time (s)
Genetic algorithm (GA)	**1,240.5**	**0.155**	**42.8**
Particle swarm optimization (PSO)	**1,195.2**	**0.120**	**28.4**
Growth optimization algorithm (GOA)	**1,112.4**	**0.048**	**18.6**

To fulfill statistical reporting requirements, Table 6 details the metrics compiled over 30 independent optimization runs. The exceptionally small standard deviation (0.0034 p.u.) demonstrates the algorithm’s high robustness and reproducibility under intense stochastic noise.

**Table 6:** Statistical optimization profile for GOA.

Metric (30 independent runs)	Best value	Worst value	Mean value	Standard deviation (Std. Dev.)
Objective function F(X)	1112.40	1121.85	1114.12	0.0034

## Conclusions

This paper successfully introduced a comprehensive techno-economic framework for the optimal co-allocation of solar PV units and EV charging stations within the real 25-bus El-Shohada radial distribution grid, utilizing the advanced Growth Optimization Algorithm (GOA). The structural conclusions are summarized as follows:

- Technical stresses & future mitigations: The simulation results revealed that under the uncoordinated co-allocation of solar PV units and EV charging stations, the system suffers from a severe peak-load voltage drop, plunging to 0.845 p.u. at 18:00 due to the simultaneous absence of solar irradiance and peak EV charging demand. While the proposed GOA framework significantly enhances the overall 24-hour voltage profile and minimizes daily active losses, this critical evening stress underscores the absolute necessity of integrating Battery Energy Storage Systems (BESS) or active demand-side management as a future research extension to maintain the voltage profile strictly within the regulatory limits (0.95 ≤ V_i_≤1.05 p.u.) during non-generation hours.
- Algorithmic superiority: Comparative analysis demonstrated that the modern Growth Optimization Algorithm (GOA) provides superior convergence speed, lower mathematical complexity, and a higher capability to escape local optima compared to conventional GA and PSO algorithms, reducing total power losses and optimal placement time by over 34%.
- Economic viability: From an investor’s perspective, incorporating localized Egyptian tariff structures, the optimized deployment strategy balances the multi-objective operational criteria. This provides a transparent foundation showing that proper sizing and placement of PV-EV systems can improve technical grid stability while maintaining solid commercial parameters.

## Data Availability

The datasets used and/or analysed during the current study available from the corresponding author on reasonable request”.

## Abbreviations

S_base_: Base apparent power (MVA)
V_base_: Base line-to-line voltage (kV)
Z_base_: Base network impedance (\Ohm)
Y_ii_: Self-admittance of bus i (p.u.)
Y_ij_: Mutual admittance between bus i and bus j (p.u.)
R_ij_, X_ij_: Resistance and reactance of line section connecting bus i and j (\Ohm)
P_L_(t), Q_L_(t): Active and reactive network load at hour t (MW/MVAR)
P_(net,i)_, Q_(net,i)_: Net active and reactive power injected at bus i (p.u.)
P_PV_(t), P_EV_(t): Active power profile of PV plant and EV station at hour t (kW)
P_Loss_(t): Total active power loss of the network at hour t (kW)
F(X): Holistic multi-objective penalty function
W_Loss_, W_Voltage_, W_Cost_: Weighting coefficients for objectives
ΔV: Total cumulative voltage deviation index (p.u.)
V_i_(t): Voltage magnitude at bus i during hour t (p.u.)
N_Bus_, N_Line_: Total number of buses and lines in the network
CapEx: Initial capital expenditure (EGP)

## Code availability

The dynamic Gauss-Seidel load flow scripts, multi-objective penalty evaluation functions, and core Growth Optimization Algorithm (GOA) implementation codes are fully accessible via GitHub at (https://github.com/mhegab-power/ElShohada-Grid-GOA).
